# Loading of osseointegrated implants for bone conduction hearing at 3 weeks: 3-year stability, survival, and tolerability

**DOI:** 10.1007/s00405-015-3746-y

**Published:** 2015-08-14

**Authors:** Rik C. Nelissen, Christine A. den Besten, Hubert T. Faber, Catharina A. J. Dun, Emmanuel A. M. Mylanus, Myrthe K. S. Hol

**Affiliations:** Department of Otorhinolaryngology, Donders Institute for Brain, Cognition and Behaviour, Radboud University Medical Center, P.O. Box 9101, 6500 HB Nijmegen, The Netherlands; Department of Otorhinolaryngology/Head and Neck Surgery, University of Groningen, University Medical Center Groningen, Groningen, The Netherlands

**Keywords:** Bone-anchored hearing aid, Baha, Early loading, ISQ, Skin reaction, Hearing loss

## Abstract

The objective of this study was to ascertain the long-term safety of loading osseointegrated implants for bone conduction hearing 3 weeks post-surgery. Thirty consecutive adult patients were implanted with the Baha BI300 (Cochlear Bone Anchored Solutions) in our tertiary referral center. Implants were loaded with the sound processor 3 weeks post-surgery. Follow-up examinations were performed at 10 days; 3, 4, 6, 8, and 12 weeks; 6 months; and 1, 2, and 3 years after implant surgery. At each follow-up visit, implant stability quotient (ISQ) values were recorded by means of resonance frequency analysis, and soft tissue status was evaluated according to Holgers’ classification. ISQ trends, implant survival, and soft tissue reactions were compared to a population of 52 patients with the same type of implants loaded from 6 weeks post-surgery as part of another study. Subjective benefit was measured by means of the Glasgow Benefit Inventory (GBI). After an initial dip in ISQ at 10 days after implantation, a gradually increasing trend in ISQ was found until 6 months in both populations, after which ISQ values remained above baseline values. Implant survival was 97 % in the study population and 96 % in the comparison population. Clinically relevant soft tissue reactions were found in 0.9 % (study population) and 1.7 % (comparison population) of all visits. Patients reported subjective benefit; the mean GBI score was 22.8. In conclusion, loading these implants at 3 weeks post-surgery is safe based on the current study, as long-term results show high ISQ values and good implant survival and tolerability.

## Introduction

Percutaneous osseointegrated titanium implants in the temporal bone have been used since 1977 to attach a vibrating sound processor to accomplish hearing amplification for several indications [1, 2]. Obviously, an implant needs to be sufficiently fixated to the bone before loading it with the sound processor is feasible. In the earliest days, implant surgery consisted of two stages, allowing a minimum of 3 months of osseointegration time before the percutaneous abutment was connected to the implant, which allowed loading with the sound processor [1, 3]. Later on, clinical application of a one-stage surgical technique was reported with a healing time of 6–8 weeks before loading the implant [4].

To allow patients to start using their device as soon as possible, yet safely, after implantation, loading times have gradually decreased. This was mostly stimulated by dental research where earlier (and even immediate) loading protocols are common practice. A recent Cochrane review [5] concluded that there was no convincing evidence of a clinically important difference in prosthesis failure, implant failure, or bone loss associated with different loading times of dental implants. However, the quality of the evidence was assessed as very low due to risks of bias in primary studies and some evidence of reporting bias. Furthermore, dental implants are different from implants for bone conduction hearing. Therefore, clinicians are advised to treat these findings with caution.

In 2005, a loading time of 4–6 weeks after implantation was advised based on expert opinion [2]. Subsequently, even earlier loading protocols have been reported [6, 7]. These results were all found using 3.75-mm-diameter implants. In 2010, a 4.5-mm-diameter implant with a moderately roughened surface was introduced. The larger implant diameter increases the bone–implant contact surface, which should theoretically result in a larger area for osseointegration, while the moderately roughened surface is thought to stimulate the initial healing response of the bone directly after implantation. Promising short-term clinical results of applying a loading time of 6 weeks after implantation with this implant were reported [8]. Subsequently, even shorter loading times of 4 [9], 3 [10], and 2 weeks [11] were advocated when using this implant.

The current study is a continuation of the study that presented the clinical results of loading at 3 weeks with a follow-up period of 6 months [10]. Study outcomes concern the clinical results on implant stability quotient (ISQ), implant survival, and soft tissue tolerability of this wide and moderately roughened implant loaded at 3 weeks with a follow-up period of 3 years. This is considerably longer than all other early loading data published to date, which have been presented with a follow-up of 1 year or less. Additionally, outcomes were compared to those from another study of the same type of implant, which, however, had been loaded from 6 weeks with a 3-year follow-up period [12]. Furthermore, subjective benefit of the bone conduction hearing system was measured [13].

## Materials and methods

### Ethical considerations

The current study was conducted in accordance with the guidelines established in the Declaration of Helsinki and the ISO 14155:2011 *Clinical investigation of medical devices for human subjects*—*Good clinical practice*, and was approved by the local ethics committee.

### Patients and implants

Thirty consecutive patients (referred to as the “study population”) were included in this prospective cohort study to have their Cochlear^®^ Baha^®^ BI300 4-mm implant with a BA300 6-mm abutment (Cochlear Bone Anchored Solutions AB, Mölnlycke, Sweden) loaded with the sound processor at 3 weeks after surgery.

Since at the conception of the current study neither clinical outcomes with the study implant nor implant loading at 3 weeks had been reported, only adult patients with normal bone quality were considered for participation in the study. To be included in the study, subjects had to meet each of the following inclusion criteria: be 18 years of age or older; be eligible for implantation and for the sound processor; and must provide written informed consent to participate in the study. Exclusion criteria were: being unable to follow the investigational procedures; simultaneous participation in another investigation with pharmaceuticals and/or devices; disease and/or treatment that compromises/will compromise the bone quality at the implant site, such as radiation therapy and osteoporosis (assessed by medical history); psychosocial problems or psychiatric disease; and, finally, the inability to attend all scheduled follow-up visits. Furthermore, if patients were assessed during implantation to have a bone thickness at the implant site of less than 4 mm, they were excluded. All subjects were free to discontinue participation in the investigation at any time without giving a reason and without prejudice regarding further treatment.

The results from the current investigation were compared to those of 52 patients (referred to as the “comparison population”), implanted with the same type of implant and abutment, reported on in a previous multicenter study with similar inclusion and exclusion criteria and an almost identical study protocol, however, with implant loading from 6 weeks after surgery. Thus, this population is not a formal control group. Therefore, methods and outcomes concerning this group will not be described in detail in the current manuscript. For more details on methods and results concerning the comparison population, please refer to the original study [8, 12].

### Study design

The primary objective of the current study was to evaluate the long-term stability of the implant placed in one-stage surgery and loaded after 21 days of healing. Secondary objectives were to evaluate the long-term survival of the implant, demonstrate the safety of the implant as assessed by the occurrence of adverse soft tissue reactions, evaluate changes in quality of life post-implantation, and, finally, evaluate hearing loss-associated disability and the reduction of disability achieved with the device. The outcomes for the study population in the present prospective investigation were compared to those of the comparison population for all study parameters that were equivalent between studies.

No sample size calculation was conducted, as no differences between groups were expected. Thus, the study population size was chosen empirically.

After inclusion and having provided written informed consent, patients in the current investigation underwent one-stage implant surgery with subcutaneous tissue reduction, according to the Nijmegen linear incision technique [14], in June and July 2010. Surgery was performed by two experienced implant surgeons (E.M. and M.H.). Follow-up examinations were performed at 10 days; 3, 4, 6, 8, and 12 weeks; 6 months; and 1, 2, and 3 years after implant surgery.

During all follow-up visits, the implant stability quotient (ISQ) was measured by means of resonance frequency analysis (RFA) with the Osstell Mentor (Osstell AB, Göteborg, Sweden), an objective and non-invasive technique. RFA provides information about the stiffness of the implant–bone junction [15] and produces two ISQ values: ISQ High and ISQ Low, usually obtained from perpendicular measurements and generally differing a few points. Both values may be used for interpretation; however, as trends should be analyzed, it is important to use one of both values consistently. In the current study, the ISQ High values were used in the statistical analyses. In cases where an abutment was replaced by a longer one during follow-up, ISQ values were no longer included in the analysis from that point on, as a change in the length of the abutment affects the ISQ values. Soft tissue status was monitored and graded according to Holgers’ classification [16].

Implant loading occurred at 3 weeks, provided that the stability of the implant (evaluated clinically and not based on an absolute ISQ value) and soft tissue status were judged to be satisfactory. Significant postoperative wound healing complications or a soft tissue reaction corresponding to a Holgers grade 3 or higher would result in postponed implant loading.

To measure subjective benefit in a standardized and comparable manner, the study population was asked to fill out the Glasgow Benefit Inventory (GBI) at 3 months and at 3 years. The GBI measures the change in health status effected by otorhinolaryngological surgical interventions [13]. Three subsections comprise 18 items: 12 relating to general improvement, 3 to social improvement, and 3 to physical improvement. Each question was answered on a five-point Likert scale. The GBI was not included in the study protocol of the comparison population’s original study that had already started more than a year earlier; hence, comparison between studies in terms of subjective benefit was not possible.

### Statistical analysis

All study data were directly entered into an SPSS data file from the patients’ medical records by the investigators. After anonymization, the data were analyzed by independent biostatisticians (Statistiska Konsultgruppen, Göteborg, Sweden). The statistical analyses were performed according to a pre-defined statistical analysis plan. A correction factor was developed and validated in the reference material by Osstell AB to transfer the ISQ values measured in the present study to corrected ISQ values to address the use of different SmartPegs, as a change in SmartPeg type was advised during the course of the study. Only these corrected ISQ values (i.e., comparable to measurements with SmartPeg type 55) are presented throughout the study to make it possible to compare ISQ values between the present study and the comparison population. A weighted average of ISQ values during the period between baseline (time of implantation) and the 3-year follow-up was obtained by determining the mean area under the curve (AUC) using the trapezoid rule. The mean AUC was calculated for the time the implant was functional. For patients who were lost to follow-up, the last observation carried forward was used in the mean AUC calculations. Comparisons between the study and comparison populations were made using Fisher’s exact test for gender, the Mann–Whitney *U* test for age and ISQ values, and the Mantel–Haenszel Chi-square test for the comparison of Holgers’ grades. A significance level of 95 % was adopted.

## Results

### Patients

A total of 31 patients were approached for participation. One patient declined participation due to logistical reasons that prohibited being available for all follow-up visits. Table 1 displays the characteristics of the 30 consecutive patients included in the study population. The characteristics of the 52 patients from a previous investigation [12], who served as the comparison population, are displayed in the same table and are found to be comparable. In both groups, three patients were excluded during follow-up. The reasons for exclusion from the current study were: implant loss (after 3 days), non-implant-related death (after 22 months), and lack of follow-up (patient left out of analyses; missed four out of eight scheduled visits).

**Table 1:** Patient characteristics for the study population (3-week loading) and the comparison population (6-week loading)

	3-week loading (*n* = 30)	6-week loading (*n* = 52)	*p*
Gender, *n* (%)			
Male	14 (46.7)	23 (44.2)	1.00
Female	16 (53.3)	29 (55.8)	
Age (years), mean (SD)	55.3 (12.3)	55.5 (13.8)	0.92
Indication for implant			
CMHL	19 (63.3)	34 (65.4)	
SSD	10 (33.3)	17 (32.7)	
Other	1 (3.3)	1. (1.9)	

*CMHL* conductive or mixed hearing loss, *SSD* single-sided sensorineural deafness

### Implant loading

Due to the implant loss that occurred before loading in one patient, 29 implants were loaded. The mean loading time in the study population was 3.2 weeks. Loading occurred in 28 patients within the 3-week visit window (mean 22 days). In one patient, loading was postponed to 36 days post-implantation because of incomplete soft tissue healing at 3 weeks post-implantation. At the 3-week follow-up, ISQ High values ranged between 64 and 73. The mean loading time in the comparison population was 8.3 weeks.

### Implant stability quotient

**Fig. 1:**
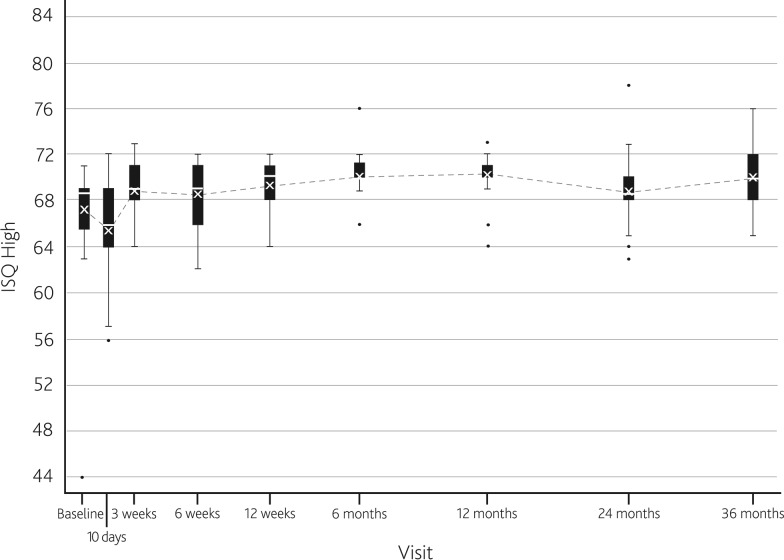
*Box* *and* *whisker plot* of ISQ High values for the study population (3-week loading). Mean (*cross*) and median (*horizontal line*) are defined within the *box plot*. *Dots* represent outlier values

**Fig. 2:**
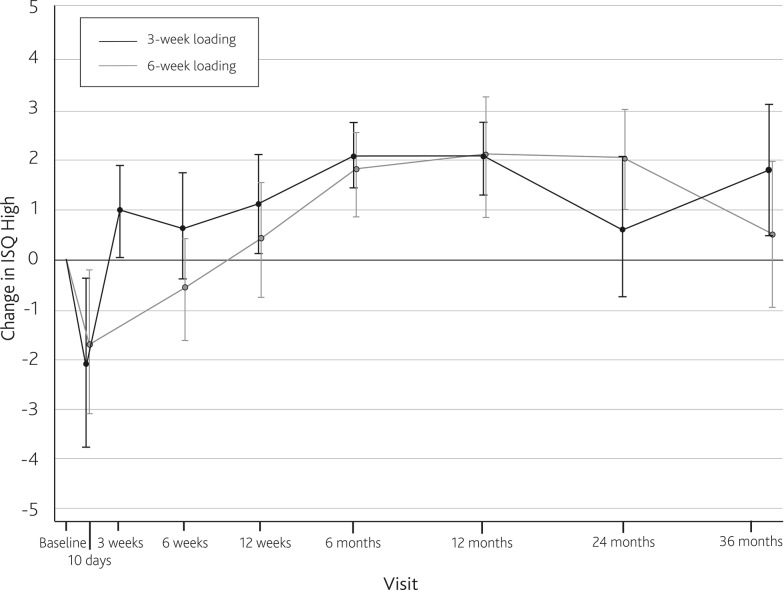
Change in ISQ High from baseline for the study population (3-week loading) and the comparison population (6-week loading)

The mean AUC for ISQ High over 3 years was 68.5 (SD 5.0) for the study population. Figure 1 displays the mean ISQ High values at each follow-up visit. After an initial dip in mean ISQ 10 days after implantation, ISQ gradually increased until 6 months after implantation. A dip in ISQ was found at 2 years, after which mean ISQ increased at 3 years to the same mean value as found at 1 year. From 2 years and onward, the spread in ISQ values increases (as seen by an increase in 95 % confidence intervals of mean ISQ). Two patients required an abutment replacement to a longer 9-mm abutment, due to soft tissue problems with the shorter 6-mm abutment. Their ISQ data were not analyzed from that point on, as different abutment lengths affect the ISQ. The trend of ISQ values as a change from baseline is similar to that observed in the comparison population, as represented by Fig. 2.

### Implant survival

Implant survival was 97 %. The sole implant loss occurred 3 days after surgery in a 65-year-old male with single-sided sensorineural deafness who had no history of diabetes or smoking. The measured ISQ at the time of implantation was 44. In the comparison population, a single implant was lost and another implant was electively removed, resulting in 96 % survival.

### Soft tissue tolerability

**Fig. 3:**
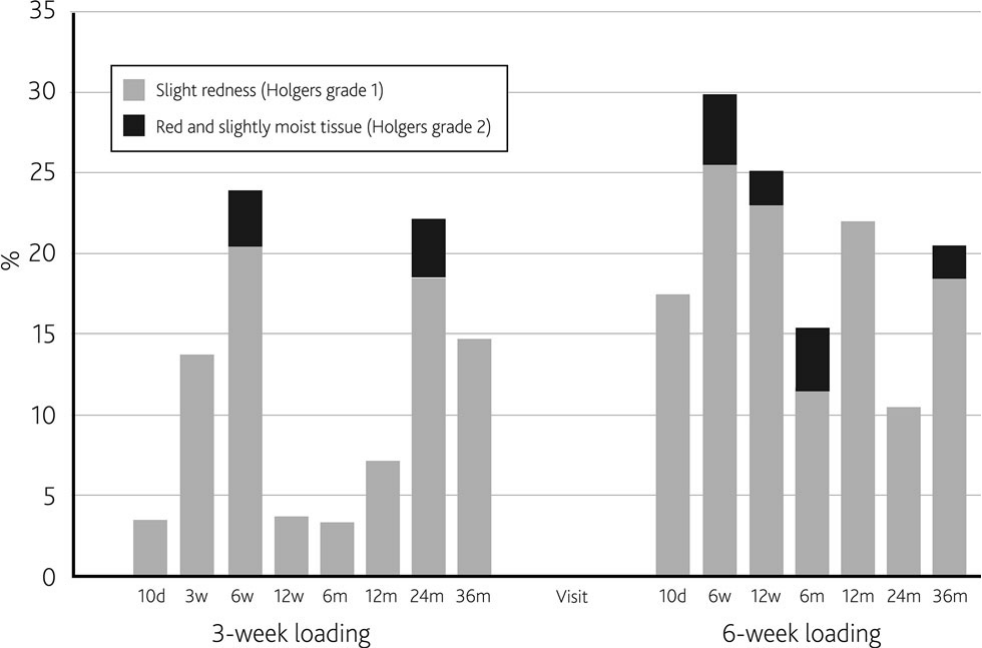
Soft tissue reactions as a percentage of visits according to Holgers’ classification for the study population (3-week loading) and the comparison population (6-week loading)

Figure 3 provides an overview of soft tissue reactions per visit. Mean local soft tissue status according to Holgers for all visits throughout the entire follow-up period were recorded: Holgers grade 0 in 88.5 %, Holgers grade 1 in 10.6 %, Holgers grade 2 in 0.9 %. No Holgers grade 3 or 4 soft tissue reactions were recorded. Similarly in the comparison population, only Holgers grade 0 (79.9 %), 1 (18.3 %), and 2 (1.7 %) were recorded. The maximum severity of soft tissue reactions throughout all visits for each patient showed no statistically significant difference (*p* = 0.22) between the study and comparison populations. Within the comparison population, the percentages of Holgers grade 2 soft tissue reactions were comparable between the different centers, each of which applied their customary soft tissue handling technique [12].

### Subjective benefit

The response rate of patients who returned a completed GBI questionnaire at the 3-year follow-up was 88.9 % (*n* = 24). Of these responders, 18 used the sound processor daily, 4 sporadically, 2 stopped, and 2 had lost their sound processors. The daily users reported a mean GBI total score of 22.8, compared to 11.1 reported by the sporadic users and −20.8 by the non-users.

## Discussion

The time between surgery and the point at which implants are loaded with the sound processor has gradually decreased over the years with the aim of enabling patients to benefit from their hearing device as quickly as possible. However, early loading should be safe and, thus, should not compromise implant stability and wound healing. The current study investigated the long-term safety of reducing the loading time to 3 weeks after implantation and provides a comparison to previously reported data on loading from 6 weeks [12]. In terms of ISQ values, implant survival and soft tissue tolerability, no significant differences were found between the two populations.

The major strength of the current study was its prospective design. Therefore, the current study data are considered to provide a substantial amount of information on the long-term safety of this early loading protocol. There is a limitation in the comparison with a group loaded from 6 weeks from another prospective multicenter study [8, 12], in that this comparison was not set up as a randomized controlled prospective trial. Furthermore, 24 (46.2 %) of the implants of the comparison population were placed using different soft tissue reduction techniques, due to the multicenter design of that original study; the use of different soft tissue reduction techniques could have led to potentially different soft tissue outcomes between the two studies (although in the multicenter study, the outcomes were shown to be comparable between sites using different techniques), but are not expected to have a significant effect on the implant stability, which was the primary analysis of the current and the former study. The inclusion and exclusion criteria of both studies were similar.

ISQ trends over time were not found to be influenced by loading the implant at 3 weeks compared to loading from 6 weeks. An initial dip in ISQ during the first follow-up examination after implantation was found, which is believed to be attributed to normal bone remodeling characteristics [10]. No decrease in ISQ values during the immediate period after implant loading at 3 weeks was observed. This suggests that the level of stability at 3 weeks after implantation of the current implant is adequate to support the sound processor. As with the comparison population, ISQ values remained above baseline values, as measured at implantation, from the time of loading until follow-up had been conducted for 3 years. Interestingly, in the study population there is a dip in mean ISQ after 2 years of follow-up, after which mean ISQ increases again at 3 years. We have no explanation for this one-point dip, but it is not deemed to be of clinical significance, as it did not result in implant loss and the ISQ values increased again at 3 years. It might be interesting to follow these implants even longer. The increase in 95 % confidence intervals of mean ISQ after 2 years is also observed in the comparison population. Furthermore, the comparison population shows a decreasing trend in ISQ at 3 years. We do not have an explanation for this decreasing trend, although a possible explanation could be marginal bone loss around the implant, a phenomenon known to occur in dental implantology [17]. However, in that case we do not understand why there is a difference between the trends for these two groups.

Despite slightly decreasing ISQ trends during some periods, implants remained clinically stable and without any peri-implant problems. The mean AUC of ISQ was slightly lower in the study population (68.5, SD 5.0) compared to the comparison population (71.5, SD 2.2). It is unclear whether this difference is clinically relevant. In the sole implant that was lost in the study population, a remarkably low ISQ value of 44 was measured at implantation. Looking at the data of the current study and other studies using implants and abutment of the same type and size [9, 11, 12], ISQ in this type of implant rarely drops below a value of 55. However, it is not currently possible to determine strict ISQ values that indicate the point at which loading is safe; only ISQ trends over time should be interpreted for clinical use. More ISQ data will be needed to establish the clinical relevance of specific values. Dental implantological studies have shown that high ISQ values are indicative of a successful implant treatment with a small risk of future failure, while low or decreasing ISQ values point to an increased risk of implant complications [15].

Implant survival was high in both the study (97 %) and comparison populations (96 %) at 3 years post-surgery, although neither of the studies was powered to demonstrate significant differences in implant survival. These figures compare positively to those reported on the previous 3.75-mm-wide as-machined titanium implants in a long-term retrospective study (92 %), which also demonstrated that implant loss occurred most frequently (79.8 % of all lost implants in that study) in the first 3 years after implantation [18]. In dental implantology, wider implant designs have been reported to increase implant stability [19] and moderately roughened surfaces have been reported to increase bone response after implantation [20].

Soft tissue tolerability was comparable between the study group and the comparison group. This indicates that earlier loading of the implant does not influence soft tissue healing negatively nor positively. The percentages of clinically relevant soft tissue reactions (Holgers grade 2 or higher) are favorable for both the study and comparison populations compared to those reported in retrospective studies [18, 21]. As these retrospective studies predominantly report on the previous implant with a conically shaped abutment, the positive soft tissue tolerability recorded in the current investigation can most likely be attributed to the new rounded abutment design, possibly in combination with a more stable implant. This is confirmed by a previous prospective randomized controlled clinical comparison of the rounded abutment design and a conically shaped abutment [12].

To date, few studies have reported long-term clinical outcomes from prospective studies of the current implant. However, long-term stability and survival are crucial parameters for a successful implant. Therefore, in addition to the need for more long-term data to be published, follow-up periods exceeding 3 years will be of great interest.

Subjective benefit as measured by the GBI in the daily users group (22.8) was comparable to the score measured at 3 months (20.9). Satisfaction with the implant and device did not change appreciably over time. As can be expected, patients who used their sound processors less frequently or not at all reported lower scores. Because the GBI was only completed by the study population, a comparison of satisfaction between both populations to establish whether earlier loading provides more subjective benefit was not possible. The currently reported GBI score corresponded to GBI scores measured in comparable study populations [22, 23].

## Conclusion

The reported long-term results of an early loading protocol at 3 weeks post-implantation indicate good ISQ values over time, implant survival, and soft tissue tolerability. These results were compared to those of the same implant type loaded from 6 weeks post-implantation in a previous investigation, which revealed no notable differences. Based on the current results, loading the tested implant is safe in adult patients with normal bone quality from 3 weeks post-implantation.
